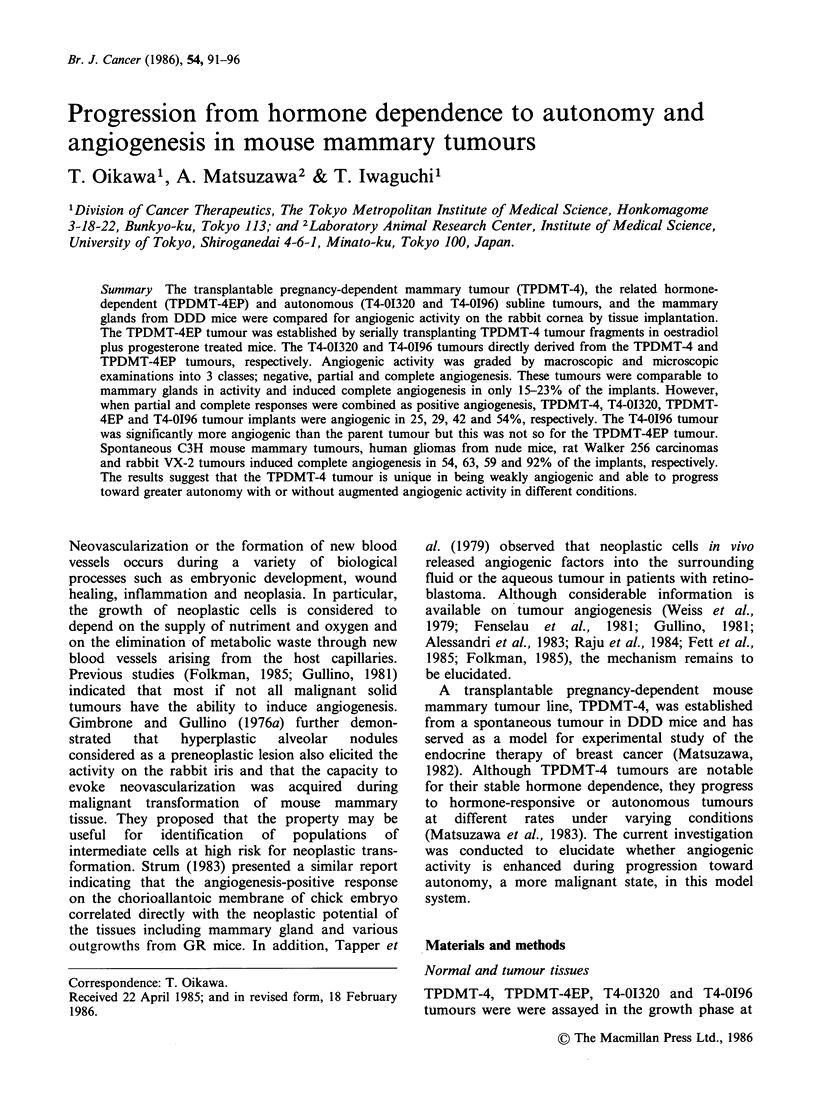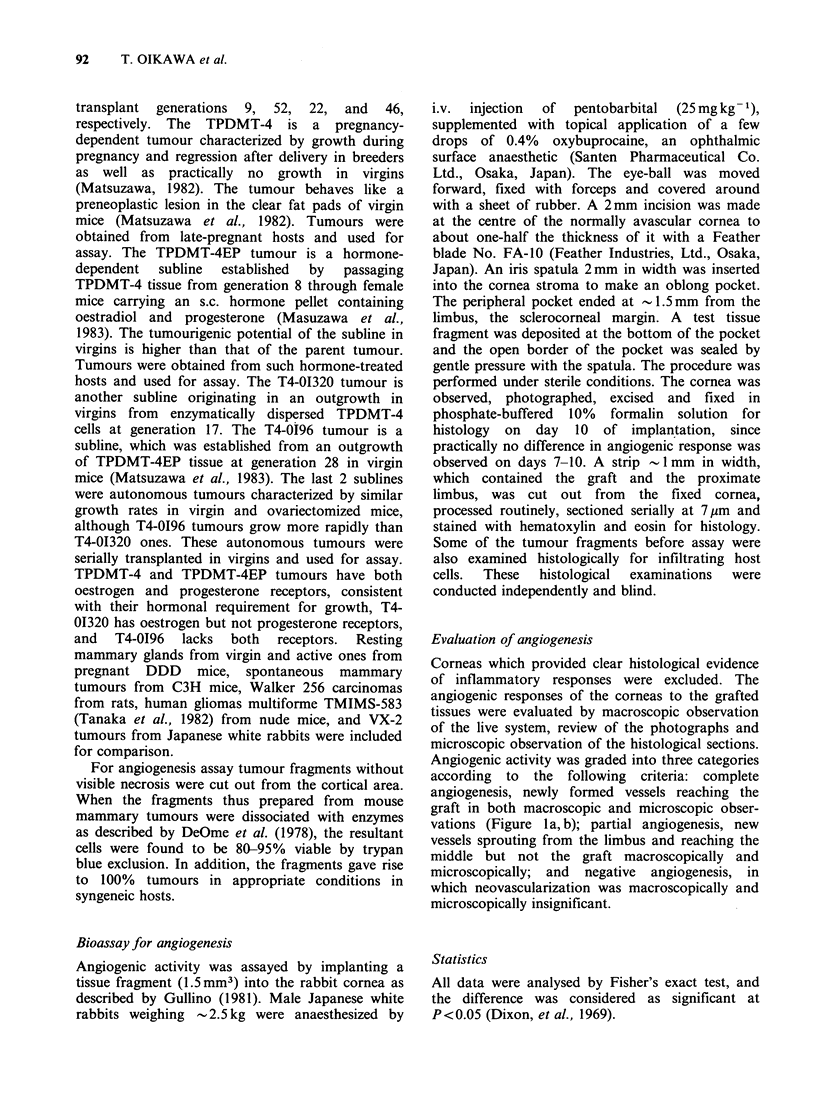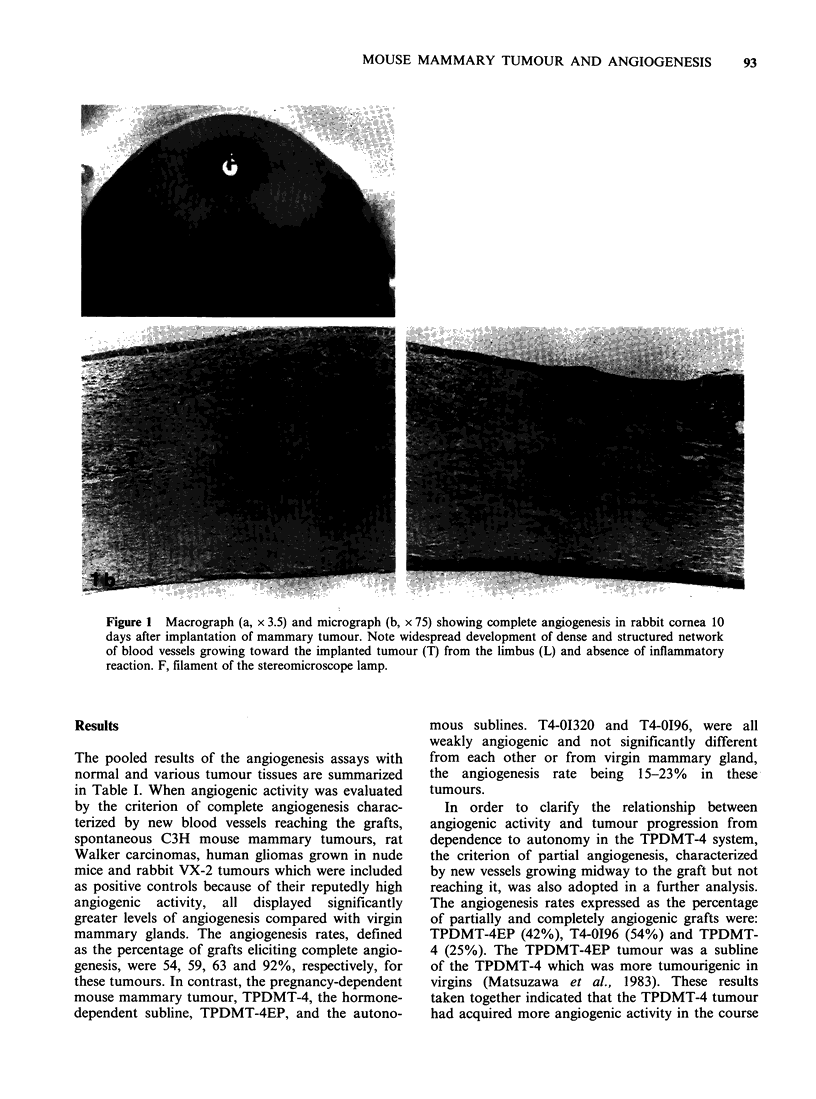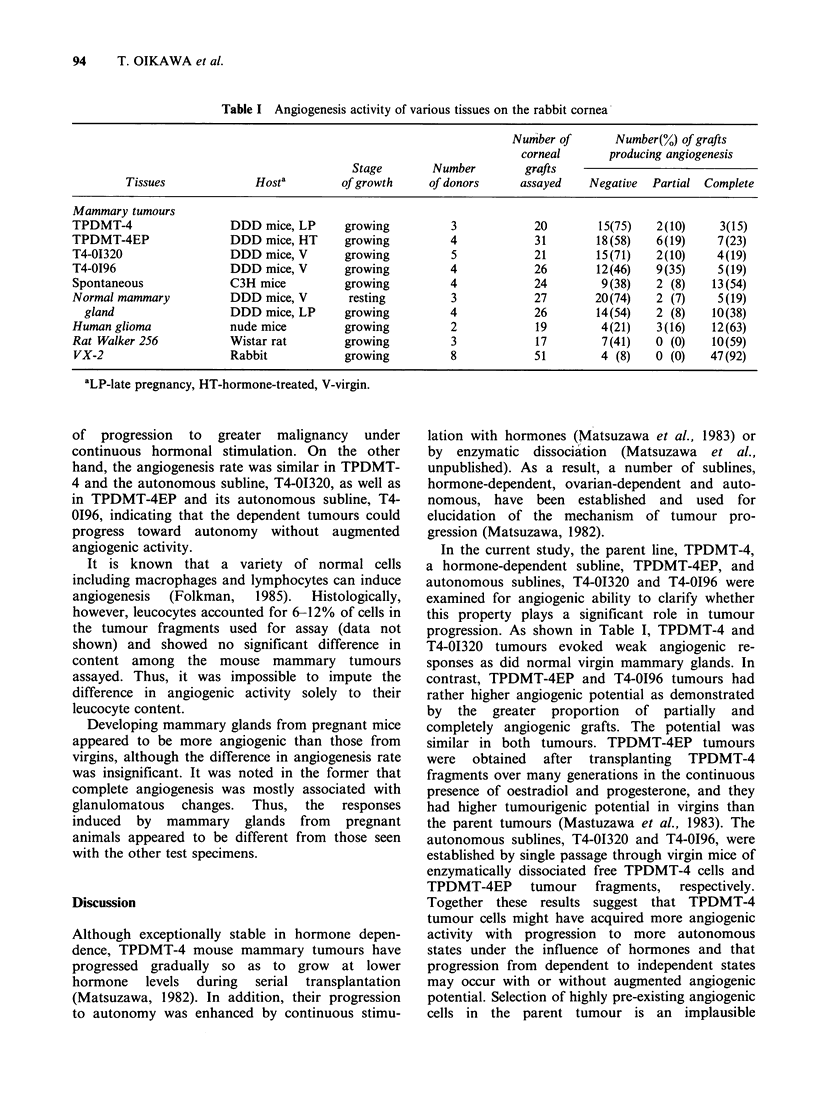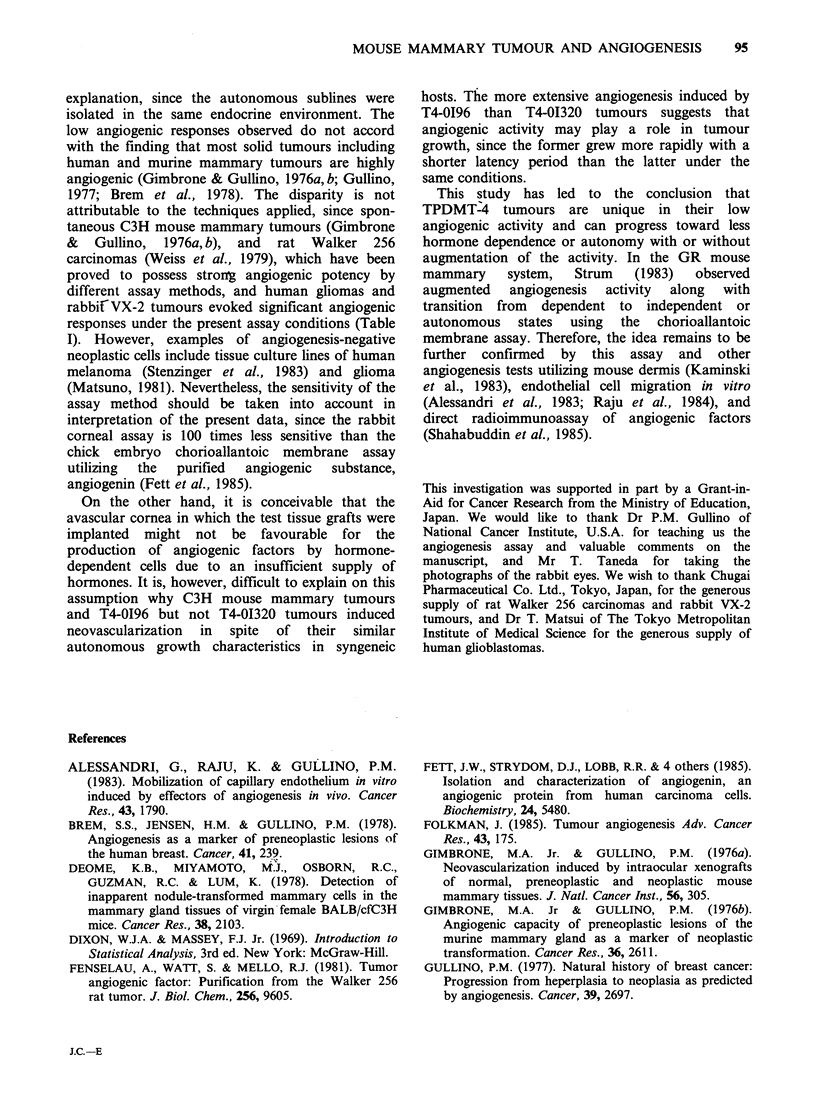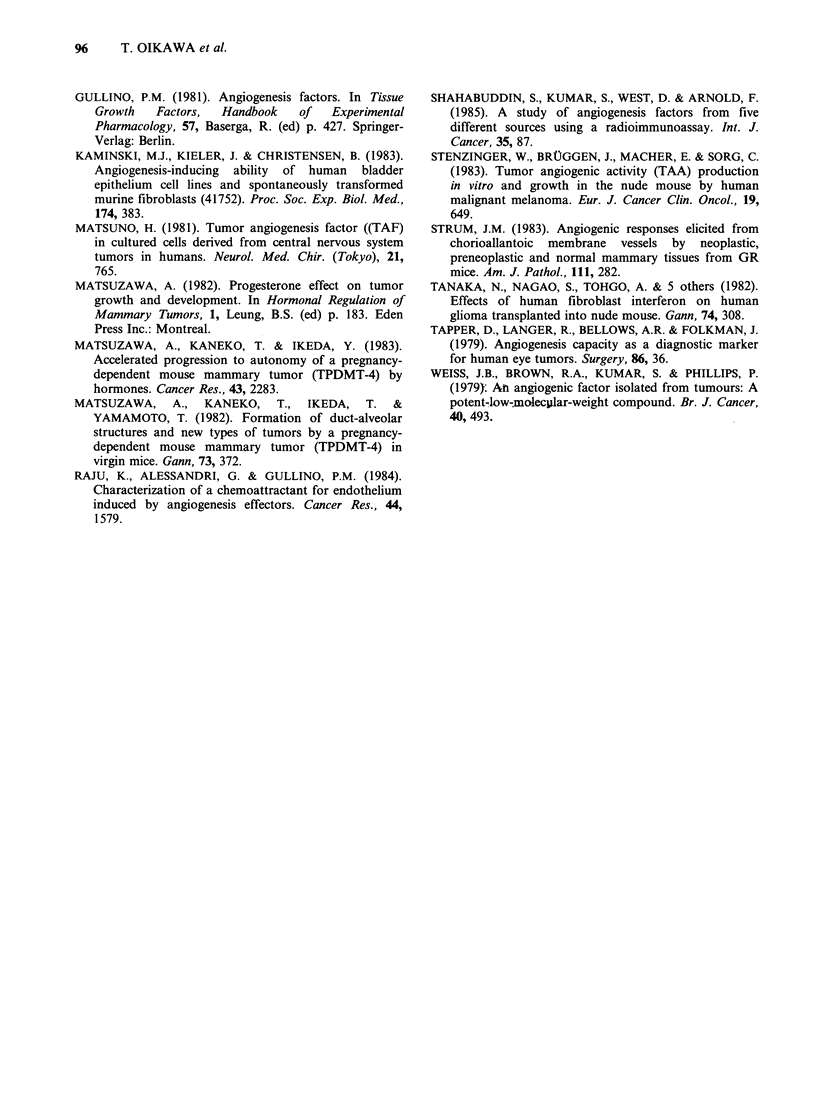# Progression from hormone dependence to autonomy and angiogenesis in mouse mammary tumours.

**DOI:** 10.1038/bjc.1986.156

**Published:** 1986-07

**Authors:** T. Oikawa, A. Matsuzawa, T. Iwaguchi

## Abstract

**Images:**


*Br. J. Cancer* (1986), **54**, 91–96

### Progression from hormone dependence to autonomy and angiogenesis in mouse mammary tumours

T. Oikawa[1], A. Matsuzawa[2] & T. Iwaguchi[1]

[1]*Division of Cancer Therapeutics, The Tokyo Metropolitan Institute of Medical Science, Honkomagome 3-18-22, Bunkyo-ku, Tokyo 113; and* [2]*Laboratory Animal Research Center, Institute of Medical Science, University of Tokyo, Shiroganedai 4-6-1, Minato-ku, Tokyo 100, Japan.*

*Summary* The transplantable pregnancy-dependent mammary tumour (TPDMT-4), the related hormone-dependent (TPDMT-4EP) and autonomous (T4-0I320 and T4-0I96) subline tumours, and the mammary glands from DDD mice were compared for angiogenic activity on the rabbit cornea by tissue implantation. The TPDMT-4EP tumour was established by serially transplanting TPDMT-4 tumour fragments in oestradiol plus progesterone treated mice. The T4-0I320 and T4-0I96 tumours directly derived from the TPDMT-4 and TPDMT-4EP tumours, respectively. Angiogenic activity was graded by macroscopic and microscopic examinations into 3 classes; negative, partial and complete angiogenesis. These tumours were comparable to mammary glands in activity and induced complete angiogenesis in only 15–23% of the implants. However, when partial and complete responses were combined as positive angiogenesis, TPDMT-4, T4-0I320, TPDMT-4EP and T4-0I96 tumour implants were angiogenic in 25, 29, 42 and 54%, respectively. The T4-0I96 tumour was significantly more angiogenic than the parent tumour but this was not so for the TPDMT-4EP tumour. Spontaneous C3H mouse mammary tumours, human gliomas from nude mice, rat Walker 256 carcinomas and rabbit VX-2 tumours induced complete angiogenesis in 54, 63, 59 and 92% of the implants, respectively. The results suggest that the TPDMT-4 tumour is unique in being weakly angiogenic and able to progress toward greater autonomy with or without augmented angiogenic activity in different conditions.

Neovascularization or the formation of new blood vessels occurs during a variety of biological processes such as embryonic development, wound healing, inflammation and neoplasia. In particular, the growth of neoplastic cells is considered to depend on the supply of nutriment and oxygen and on the elimination of metabolic waste through new blood vessels arising from the host capillaries. Previous studies (Folkman, 1985; Gullino, 1981) indicated that most if not all malignant solid tumours have the ability to induce angiogenesis. Gimbrone and Gullino (1976*a*) further demonstrated that hyperplastic alveolar nodules considered as a preneoplastic lesion also elicited the activity on the rabbit iris and that the capacity to evoke neovascularization was acquired during malignant transformation of mouse mammary tissue. They proposed that the property may be useful for identification of populations of intermediate cells at high risk for neoplastic transformation. Strum (1983) presented a similar report indicating that the angiogenesis-positive response on the chorioallantoic membrane of chick embryo correlated directly with the neoplastic potential of the tissues including mammary gland and various outgrowths from GR mice. In addition, Tapper *et al.* (1979) observed that neoplastic cells *in vivo* released angiogenic factors into the surrounding fluid or the aqueous tumour in patients with retinoblastoma. Although considerable information is available on tumour angiogenesis (Weiss *et al.*, 1979; Fenselau *et al.*, 1981; Gullino, 1981; Alessandri *et al.*, 1983; Raju *et al.*, 1984; Fett *et al.*, 1985; Folkman, 1985), the mechanism remains to be elucidated.

A transplantable pregnancy-dependent mouse mammary tumour line, TPDMT-4, was established from a spontaneous tumour in DDD mice and has served as a model for experimental study of the endocrine therapy of breast cancer (Matsuzawa, 1982). Although TPDMT-4 tumours are notable for their stable hormone dependence, they progress to hormone-responsive or autonomous tumours at different rates under varying conditions (Matsuzawa *et al.*, 1983). The current investigation was conducted to elucidate whether angiogenic activity is enhanced during progression toward autonomy, a more malignant state, in this model system.

### Materials and methods

### *Normal and tumour tissues*

TPDMT-4, TPDMT-4EP, T4-0I320 and T4-0I96 tumours were were assayed in the growth phase at

Correspondence: T. Oikawa.
Received 22 April 1985; and in revised form, 18 February 1986.

© The Macmillan Press Ltd., 1986

transplant generations 9, 52, 22, and 46, respectively. The TPDMT-4 is a pregnancy-dependent tumour characterized by growth during pregnancy and regression after delivery in breeders as well as practically no growth in virgins (Matsuzawa, 1982). The tumour behaves like a preneoplastic lesion in the clear fat pads of virgin mice (Matsuzawa *et al.*, 1982). Tumours were obtained from late-pregnant hosts and used for assay. The TPDMT-4EP tumour is a hormone-dependent subline established by passaging TPDMT-4 tissue from generation 8 through female mice carrying an s.c. hormone pellet containing oestradiol and progesterone (Masuzawa *et al.*, 1983). The tumourigenic potential of the subline in virgins is higher than that of the parent tumour. Tumours were obtained from such hormone-treated hosts and used for assay. The T4-0I320 tumour is another subline originating in an outgrowth in virgins from enzymatically dispersed TPDMT-4 cells at generation 17. The T4-0I96 tumour is a subline, which was established from an outgrowth of TPDMT-4EP tissue at generation 28 in virgin mice (Matsuzawa *et al.*, 1983). The last 2 sublines were autonomous tumours characterized by similar growth rates in virgin and ovariectomized mice, although T4-0I96 tumours grow more rapidly than T4-0I320 ones. These autonomous tumours were serially transplanted in virgins and used for assay. TPDMT-4 and TPDMT-4EP tumours have both oestrogen and progesterone receptors, consistent with their hormonal requirement for growth, T4-0I320 has oestrogen but not progesterone receptors, and T4-0I96 lacks both receptors. Resting mammary glands from virgin and active ones from pregnant DDD mice, spontaneous mammary tumours from C3H mice, Walker 256 carcinomas from rats, human gliomas multiforme TMIMS-583 (Tanaka *et al.*, 1982) from nude mice, and VX-2 tumours from Japanese white rabbits were included for comparison.

For angiogenesis assay tumour fragments without visible necrosis were cut out from the cortical area. When the fragments thus prepared from mouse mammary tumours were dissociated with enzymes as described by DeOme *et al.* (1978), the resultant cells were found to be 80–95% viable by trypan blue exclusion. In addition, the fragments gave rise to 100% tumours in appropriate conditions in syngeneic hosts.

*Bioassay for angiogenesis*

Angiogenic activity was assayed by implanting a tissue fragment ($1.5\,mm^3$) into the rabbit cornea as described by Gullino (1981). Male Japanese white rabbits weighing $\sim$2.5 kg were anaesthesized by i.v. injection of pentobarbital ($25\,mg\,kg^{-1}$), supplemented with topical application of a few drops of 0.4% oxybuprocaine, an ophthalmic surface anaesthetic (Santen Pharmaceutical Co. Ltd., Osaka, Japan). The eye-ball was moved forward, fixed with forceps and covered around with a sheet of rubber. A 2 mm incision was made at the centre of the normally avascular cornea to about one-half the thickness of it with a Feather blade No. FA-10 (Feather Industries, Ltd., Osaka, Japan). An iris spatula 2 mm in width was inserted into the cornea stroma to make an oblong pocket. The peripheral pocket ended at $\sim$1.5 mm from the limbus, the sclerocorneal margin. A test tissue fragment was deposited at the bottom of the pocket and the open border of the pocket was sealed by gentle pressure with the spatula. The procedure was performed under sterile conditions. The cornea was observed, photographed, excised and fixed in phosphate-buffered 10% formalin solution for histology on day 10 of implantation, since practically no difference in angiogenic response was observed on days 7–10. A strip $\sim$1 mm in width, which contained the graft and the proximate limbus, was cut out from the fixed cornea, processed routinely, sectioned serially at 7 $\mu$m and stained with hematoxylin and eosin for histology. Some of the tumour fragments before assay were also examined histologically for infiltrating host cells. These histological examinations were conducted independently and blind.

*Evaluation of angiogenesis*

Corneas which provided clear histological evidence of inflammatory responses were excluded. The angiogenic responses of the corneas to the grafted tissues were evaluated by macroscopic observation of the live system, review of the photographs and microscopic observation of the histological sections. Angiogenic activity was graded into three categories according to the following criteria: complete angiogenesis, newly formed vessels reaching the graft in both macroscopic and microscopic observations (Figure 1a, b); partial angiogenesis, new vessels sprouting from the limbus and reaching the middle but not the graft macroscopically and microscopically; and negative angiogenesis, in which neovascularization was macroscopically and microscopically insignificant.

*Statistics*

All data were analysed by Fisher's exact test, and the difference was considered as significant at $P<0.05$ (Dixon, *et al.*, 1969).

**Figure 1** Macrograph (a, × 3.5) and micrograph (b, × 75) showing complete angiogenesis in rabbit cornea 10 days after implantation of mammary tumour. Note widespread development of dense and structured network of blood vessels growing toward the implanted tumour (T) from the limbus (L) and absence of inflammatory reaction. F, filament of the stereomicroscope lamp.

### Results

The pooled results of the angiogenesis assays with normal and various tumour tissues are summarized in Table I. When angiogenic activity was evaluated by the criterion of complete angiogenesis characterized by new blood vessels reaching the grafts, spontaneous C3H mouse mammary tumours, rat Walker carcinomas, human gliomas grown in nude mice and rabbit VX-2 tumours which were included as positive controls because of their reputedly high angiogenic activity, all displayed significantly greater levels of angiogenesis compared with virgin mammary glands. The angiogenesis rates, defined as the percentage of grafts eliciting complete angiogenesis, were 54, 59, 63 and 92%, respectively, for these tumours. In contrast, the pregnancy-dependent mouse mammary tumour, TPDMT-4, the hormone-dependent subline, TPDMT-4EP, and the autonomous sublines. T4-0I320 and T4-0I96, were all weakly angiogenic and not significantly different from each other or from virgin mammary gland, the angiogenesis rate being 15–23% in these tumours.

In order to clarify the relationship between angiogenic activity and tumour progression from dependence to autonomy in the TPDMT-4 system, the criterion of partial angiogenesis, characterized by new vessels growing midway to the graft but not reaching it, was also adopted in a further analysis. The angiogenesis rates expressed as the percentage of partially and completely angiogenic grafts were: TPDMT-4EP (42%), T4-0I96 (54%) and TPDMT-4 (25%). The TPDMT-4EP tumour was a subline of the TPDMT-4 which was more tumourigenic in virgins (Matsuzawa *et al.*, 1983). These results taken together indicated that the TPDMT-4 tumour had acquired more angiogenic activity in the course

**Table I** Angiogenesis activity of various tissues on the rabbit cornea

| *Tissues* | *Host*[a] | *Stage of growth* | *Number of donors* | *Number of corneal grafts assayed* | *Number(%) of grafts producing angiogenesis* | | |
|---|---|---|---|---|---|---|---|
| | | | | | *Negative* | *Partial* | *Complete* |
| *Mammary tumours* | | | | | | | |
| TPDMT-4 | DDD mice, LP | growing | 3 | 20 | 15(75) | 2(10) | 3(15) |
| TPDMT-4EP | DDD mice, HT | growing | 4 | 31 | 18(58) | 6(19) | 7(23) |
| T4-01320 | DDD mice, V | growing | 5 | 21 | 15(71) | 2(10) | 4(19) |
| T4-0196 | DDD mice, V | growing | 4 | 26 | 12(46) | 9(35) | 5(19) |
| Spontaneous | C3H mice | growing | 4 | 24 | 9(38) | 2 (8) | 13(54) |
| *Normal mammary* | DDD mice, V | resting | 3 | 27 | 20(74) | 2 (7) | 5(19) |
| *gland* | DDD mice, LP | growing | 4 | 26 | 14(54) | 2 (8) | 10(38) |
| *Human glioma* | nude mice | growing | 2 | 19 | 4(21) | 3(16) | 12(63) |
| *Rat Walker 256* | Wistar rat | growing | 3 | 17 | 7(41) | 0 (0) | 10(59) |
| *VX-2* | Rabbit | growing | 8 | 51 | 4 (8) | 0 (0) | 47(92) |

[a]LP-late pregnancy, HT-hormone-treated, V-virgin.

of progression to greater malignancy under continuous hormonal stimulation. On the other hand, the angiogenesis rate was similar in TPDMT-4 and the autonomous subline, T4-01320, as well as in TPDMT-4EP and its autonomous subline, T4-0196, indicating that the dependent tumours could progress toward autonomy without augmented angiogenic activity.

It is known that a variety of normal cells including macrophages and lymphocytes can induce angiogenesis (Folkman, 1985). Histologically, however, leucocytes accounted for 6–12% of cells in the tumour fragments used for assay (data not shown) and showed no significant difference in content among the mouse mammary tumours assayed. Thus, it was impossible to impute the difference in angiogenic activity solely to their leucocyte content.

Developing mammary glands from pregnant mice appeared to be more angiogenic than those from virgins, although the difference in angiogenesis rate was insignificant. It was noted in the former that complete angiogenesis was mostly associated with glanulomatous changes. Thus, the responses induced by mammary glands from pregnant animals appeared to be different from those seen with the other test specimens.

### Discussion

Although exceptionally stable in hormone dependence, TPDMT-4 mouse mammary tumours have progressed gradually so as to grow at lower hormone levels during serial transplantation (Matsuzawa, 1982). In addition, their progression to autonomy was enhanced by continuous stimulation with hormones (Matsuzawa *et al.*, 1983) or by enzymatic dissociation (Matsuzawa *et al.*, unpublished). As a result, a number of sublines, hormone-dependent, ovarian-dependent and autonomous, have been established and used for elucidation of the mechanism of tumour progression (Matsuzawa, 1982).

In the current study, the parent line, TPDMT-4, a hormone-dependent subline, TPDMT-4EP, and autonomous sublines, T4-01320 and T4-0196 were examined for angiogenic ability to clarify whether this property plays a significant role in tumour progression. As shown in Table I, TPDMT-4 and T4-01320 tumours evoked weak angiogenic responses as did normal virgin mammary glands. In contrast, TPDMT-4EP and T4-0196 tumours had rather higher angiogenic potential as demonstrated by the greater proportion of partially and completely angiogenic grafts. The potential was similar in both tumours. TPDMT-4EP tumours were obtained after transplanting TPDMT-4 fragments over many generations in the continuous presence of oestradiol and progesterone, and they had higher tumourigenic potential in virgins than the parent tumours (Mastuzawa *et al.*, 1983). The autonomous sublines, T4-01320 and T4-0196, were established by single passage through virgin mice of enzymatically dissociated free TPDMT-4 cells and TPDMT-4EP tumour fragments, respectively. Together these results suggest that TPDMT-4 tumour cells might have acquired more angiogenic activity with progression to more autonomous states under the influence of hormones and that progression from dependent to independent states may occur with or without augmented angiogenic potential. Selection of highly pre-existing angiogenic cells in the parent tumour is an implausible explanation, since the autonomous sublines were isolated in the same endocrine environment. The low angiogenic responses observed do not accord with the finding that most solid tumours including human and murine mammary tumours are highly angiogenic (Gimbrone & Gullino, 1976*a, b*; Gullino, 1977; Brem *et al.*, 1978). The disparity is not attributable to the techniques applied, since spontaneous C3H mouse mammary tumours (Gimbrone & Gullino, 1976*a,b*), and rat Walker 256 carcinomas (Weiss *et al.*, 1979), which have been proved to possess strong angiogenic potency by different assay methods, and human gliomas and rabbit VX-2 tumours evoked significant angiogenic responses under the present assay conditions (Table I). However, examples of angiogenesis-negative neoplastic cells include tissue culture lines of human melanoma (Stenzinger *et al.*, 1983) and glioma (Matsuno, 1981). Nevertheless, the sensitivity of the assay method should be taken into account in interpretation of the present data, since the rabbit corneal assay is 100 times less sensitive than the chick embryo chorioallantoic membrane assay utilizing the purified angiogenic substance, angiogenin (Fett *et al.*, 1985).

On the other hand, it is conceivable that the avascular cornea in which the test tissue grafts were implanted might not be favourable for the production of angiogenic factors by hormone-dependent cells due to an insufficient supply of hormones. It is, however, difficult to explain on this assumption why C3H mouse mammary tumours and T4-0I96 but not T4-0I320 tumours induced neovascularization in spite of their similar autonomous growth characteristics in syngeneic hosts. The more extensive angiogenesis induced by T4-0I96 than T4-0I320 tumours suggests that angiogenic activity may play a role in tumour growth, since the former grew more rapidly with a shorter latency period than the latter under the same conditions.

This study has led to the conclusion that TPDMT-4 tumours are unique in their low angiogenic activity and can progress toward less hormone dependence or autonomy with or without augmentation of the activity. In the GR mouse mammary system, Strum (1983) observed augmented angiogenesis activity along with transition from dependent to independent or autonomous states using the chorioallantoic membrane assay. Therefore, the idea remains to be further confirmed by this assay and other angiogenesis tests utilizing mouse dermis (Kaminski *et* al., 1983), endothelial cell migration *in vitro* (Alessandri *et al.*, 1983; Raju *et al.*, 1984), and direct radioimmunoassay of angiogenic factors (Shahabuddin *et al.*, 1985).

This investigation was supported in part by a Grant-in-Aid for Cancer Research from the Ministry of Education, Japan. We would like to thank Dr P.M. Gullino of National Cancer Institute, U.S.A. for teaching us the angiogenesis assay and valuable comments on the manuscript, and Mr T. Taneda for taking the photographs of the rabbit eyes. We wish to thank Chugai Pharmaceutical Co. Ltd., Tokyo, Japan, for the generous supply of rat Walker 256 carcinomas and rabbit VX-2 tumours, and Dr T. Matsui of The Tokyo Metropolitan Institute of Medical Science for the generous supply of human glioblastomas.

### References

ALESSANDRI, G., RAJU, K. & GULLINO, P.M. (1983). Mobilization of capillary endothelium *in vitro* induced by effectors of angiogenesis *in vivo*. *Cancer Res.*, **43**, 1790.

BREM, S.S., JENSEN, H.M. & GULLINO, P.M. (1978). Angiogenesis as a marker of preneoplastic lesions of the human breast. *Cancer*, **41**, 239.

DEOME, K.B., MIYAMOTO, M.J., OSBORN, R.C., GUZMAN, R.C. & LUM, K. (1978). Detection of inapparent nodule-transformed mammary cells in the mammary gland tissues of virgin female BALB/cfC3H mice. *Cancer Res.*, **38**, 2103.

DIXON, W.J.A. & MASSEY, F.J. Jr. (1969). *Introduction to Statistical Analysis*, 3rd ed. New York: McGraw-Hill.

FENSELAU, A., WATT, S. & MELLO, R.J. (1981). Tumor angiogenic factor: Purification from the Walker 256 rat tumor. *J. Biol. Chem.*, **256**, 9605.

FETT, J.W., STRYDOM, D.J., LOBB, R.R. & 4 others (1985). Isolation and characterization of angiogenin, an angiogenic protein from human carcinoma cells. *Biochemistry*, **24**, 5480.

FOLKMAN, J. (1985). Tumour angiogenesis *Adv. Cancer Res.*, **43**, 175.

GIMBRONE, M.A. Jr. & GULLINO, P.M. (1976*a*). Neovascularization induced by intraocular xenografts of normal, preneoplastic and neoplastic mouse mammary tissues. *J. Natl. Cancer Inst.*, **56**, 305.

GIMBRONE, M.A. Jr & GULLINO, P.M. (1976*b*). Angiogenic capacity of preneoplastic lesions of the murine mammary gland as a marker of neoplastic transformation. *Cancer Res.*, **36**, 2611.

GULLINO, P.M. (1977). Natural history of breast cancer: Progression from heperplasia to neoplasia as predicted by angiogenesis. *Cancer*, **39**, 2697.

GULLINO, P.M. (1981). Angiogenesis factors. In *Tissue Growth Factors, Handbook of Experimental Pharmacology,* **57,** Baserga, R. (ed) p. 427. Springer-Verlag: Berlin.

KAMINSKI, M.J., KIELER, J. & CHRISTENSEN, B. (1983). Angiogenesis-inducing ability of human bladder epithelium cell lines and spontaneously transformed murine fibroblasts (41752). *Proc. Soc. Exp. Biol. Med.,* **174,** 383.

MATSUNO, H. (1981). Tumor angiogenesis factor ((TAF) in cultured cells derived from central nervous system tumors in humans. *Neurol. Med. Chir.* (*Tokyo*), **21,** 765.

MATSUZAWA, A. (1982). Progesterone effect on tumor growth and development. In *Hormonal Regulation of Mammary Tumors,* **1,** Leung, B.S. (ed) p. 183. Eden Press Inc.: Montreal.

MATSUZAWA, A., KANEKO, T. & IKEDA, Y. (1983). Accelerated progression to autonomy of a pregnancy-dependent mouse mammary tumor (TPDMT-4) by hormones. *Cancer Res.,* **43,** 2283.

MATSUZAWA, A., KANEKO, T., IKEDA, T. & YAMAMOTO, T. (1982). Formation of duct-alveolar structures and new types of tumors by a pregnancy-dependent mouse mammary tumor (TPDMT-4) in virgin mice. *Gann,* **73,** 372.

RAJU, K., ALESSANDRI, G. & GULLINO, P.M. (1984). Characterization of a chemoattractant for endothelium induced by angiogenesis effectors. *Cancer Res.,* **44,** 1579.

SHAHABUDDIN, S., KUMAR, S., WEST, D. & ARNOLD, F. (1985). A study of angiogenesis factors from five different sources using a radioimmunoassay. *Int. J. Cancer,* **35,** 87.

STENZINGER, W., BRÜGGEN, J., MACHER, E. & SORG, C. (1983). Tumor angiogenic activity (TAA) production *in vitro* and growth in the nude mouse by human malignant melanoma. *Eur. J. Cancer Clin. Oncol.,* **19,** 649.

STRUM, J.M. (1983). Angiogenic responses elicited from chorioallantoic membrane vessels by neoplastic, preneoplastic and normal mammary tissues from GR mice. *Am. J. Pathol.,* **111,** 282.

TANAKA, N., NAGAO, S., TOHGO, A. & 5 others (1982). Effects of human fibroblast interferon on human glioma transplanted into nude mouse. *Gann,* **74,** 308.

TAPPER, D., LANGER, R., BELLOWS, A.R. & FOLKMAN, J. (1979). Angiogenesis capacity as a diagnostic marker for human eye tumors. *Surgery,* **86,** 36.

WEISS, J.B., BROWN, R.A., KUMAR, S. & PHILLIPS, P. (1979). An angiogenic factor isolated from tumours: A potent low-molecular-weight compound. *Br. J. Cancer,* **40,** 493.